# Effects of Meaningful Action Observation Therapy on Occupational Performance, Upper Limb Function, and Corticospinal Excitability Poststroke: A Double-Blind Randomized Control Trial

**DOI:** 10.1155/2022/5284044

**Published:** 2022-09-16

**Authors:** Aryan Shamili, Afsoon Hassani Mehraban, Akram Azad, Gholam Reza Raissi, Mohsen Shati

**Affiliations:** ^1^Rehabilitation Research Center, Department of Occupational Therapy, School of Rehabilitation Sciences, Iran University of Medical Sciences (IUMS), Tehran, Iran; ^2^Research Center for War-Affected People, Tehran University of Medical Sciences, Tehran, Iran; ^3^Neuromusculoskeletal Research Center, Iran University of Medical Sciences (IUMS), Tehran, Iran; ^4^Mental Health Research Center, School of Behavioral Sciences and Mental Health, Tehran Institute of Psychiatry, Iran University of Medical Sciences (IUMS), Tehran, Iran

## Abstract

**Introduction:**

Action observation therapy (AOT) is a mirror neuron-based approach that has been recently used in poststroke rehabilitation. The main goal of this study was to investigate the effectiveness of AOT of occupations and tasks that are meaningful for chronic stroke patients on occupational performance, upper-extremity function, and corticospinal changes.

**Method:**

A randomized control trial was designed to compare between experimental (*n* = 13) and control groups (*n* = 14). In both groups, the execution of meaningful tasks was practiced, but the videos of those tasks were just shown to the experiment group. Instead, patients in the control group watched nature videos as a placebo. Clinical outcomes were evaluated using the Canadian Occupational Performance Measure (COPM), Fugl-Meyer Assessment (FMA), Action Research Arm Test (ARAT), and Box-Block Test (BBT) on 3 occasions: baseline, post (at 4 weeks), and follow-up (at 8 weeks). The assessments of central motor conduction time (CMCT) for abductor policis brevis (APB) and extensor indicis (EI) were only recorded at baseline and posttreatment. Both assessors of clinical and neurophysiological outcomes were blinded to the allocation of subjects.

**Result:**

Finally, the results of outcomes in 24 patients who completed the study were analyzed. In both groups, significant improvements after treatment were seen for most outcomes (*p* ≤ 0.05). These changes were persistent until follow-up. There were significant differences in COPM performance (*p* = 0.03) and satisfaction (*p* = 0.001) between the experimental and control groups. In contrast, other clinical assessments such as FMA, ARAT, and BBT did not show significant differences between the two treatments (*p* ≥ 0.05). The results of CMCT related to APB showed a more significant change in the experiment group compared to the control group (*p* = 0.022). There was no difference in change detected between the two groups for CMCT related to EI after treatments.

**Conclusion:**

Observation and execution of meaningful activities can enhance the effects of simply practicing those activities on occupational performance/satisfaction and corticospinal excitability poststroke.

## 1. Introduction

Stroke is still a main cause of death and disability globally, with many healthy lives lost each year [1]. It has been reported that at 12 months after stroke, approximately 61% of patients die or become disabled [2]. After a year poststroke, many of those who live with disability face physical and/or occupational dependency (66%–75%) [3]. Participation in daily life and social activities is closely related to upper limb function [4]. About two-thirds of patients with stroke will have continued upper extremity problems for months and years [5], which reduce their participation in meaningful occupations [6]. Therefore, improving the motor and functional recovery of the upper extremities might be a key for appropriate occupational function and consequently for enhancing the quality of life poststroke [4].

Due to the fact that the results of many rehabilitation methods available after stroke are not satisfactory, conducting research with basic and clinical rationale is very important to achieve better results, especially in the field of upper limb problems [7, 8].

Action observation therapy (AOT) is a new method used in upper limb rehabilitation of various neurological disorders, especially in cerebral palsy [9] and stroke [10]. In the process of AOT, patients watch some movements and actions of healthy subjects on a video or a live show; afterwards, the patients should try to imitate and perform those actions [10]. Researches have argued that the theory behind AOT is explained by the evidence that observation of a purposeful action [11, 12] stimulates the mirror neuron system (MNS) which is the neural active mechanism, while the same action is being executed [13]. It is reported that mirror neuron areas of the brain have functional connections with the motor cortex [14, 15]. So it might be possible to change cortical motor representations as well as motor recovery of impaired limbs after stroke by the activation of MNS during the AOT process [16].

Mirror neurons form a system in the brain that has characteristics such as being (1) purposeful, (2) context-dependent, (3) experience-based, and (4) multisensory [17]. It seems that the more these features are amplified, the more likely the MNS and other brain circuits will be excited and prepared for the potential neuroplasticity. The mentioned characteristics of the MNS are inherent in many activities of patients' daily life. It is believed that the mirror neuron system is more active when observing a complex and purposeful activity compared to a simple action, so one way for more MNS excitation might be using activities that are in line with everyday activities and based on one's experiences [18, 19].

As explained above, AOT is on the basis of MNS theories, and it might be possible that by augmenting MNS function, progress will be achieved within this technique. This advantage might lead to a better motor recovery and upper limb function in relative occupations of stroke patients. On the one hand, there are studies that have investigated the effectiveness of AOT on upper limb motor function poststroke [20–23]. However, there are no considerable studies and evidence on the effects of AOT on occupation and participation areas [23] as well as effects on central nervous system neural changes [24].

On the other hand, there are many studies that have used simple movements or less purposeful tasks for observation and execution, such as finger movements and manipulating objects such as ball or blocks [22–28]. Even those studies that provided more complicated and purposeful activities during AOT, such as drinking a cup of tea and playing with coins and cards [29], did not consider the occupational priorities and meaningfulness of the activities from the patient's view. According to theories, purposefulness can promote motor learning but not all purposeful activities are meaningful and there are some differences between these two concepts [30]. Therefore, a shortage of research still remains on the use of activities/occupations which are selected by the patients and are meaningful to them. Meaningfulness is believed to make the therapy more collaborative and motivational because it originates from issues such as the client's needs, experiences, and context [31]. These issues seem to be the same as the characteristics of the MNS that was mentioned earlier. Taking into account the common characteristics of the MNS and meaningful occupations, in this study, observing and performing meaningful daily occupations close to real world was investigated whether it might be a beneficial intervention.

The main hypothesis of this study was: “observation and execution of meaningful tasks/occupations selected by the patients can enhance occupational performance/satisfaction compared to only execution of the same tasks/occupations.” We also compared the changes in motor recovery and performance of upper limb and also cortical excitability between these two interventions.

## 2. Methods

### 2.1. Participants

With regard to the aim of this study to examine meaningful AOT, determining and selecting a popular and highly important occupation among the priorities of chronic stroke patients were a necessity. According to evidence [32], the opinion of the experts, and interview with 104 available chronic stroke patients, the occupation of *eating* was recognized as an important and meaningful occupation for many of these clients; therefore, it was considered as a main inclusion criteria. The criteria for entering the study were choosing the *eating* occupation in the priority list of Canadian Occupational Performance Measure (COPM) with an importance score ≥6 out of 10, age between 40 and 70, at least 6 months poststroke, a score above 23 on the Persian version of cognitive test of Mini Mental Status Exam (MMSE) [33], no other neurological diseases, history of only 1 stroke, motor recovery stage between 3 and 5 according to Brunnstrom's classification, and no history of cranial implants or seizures. Chronic stroke patients attending rehabilitation centers or local hospitals were recruited with a convenience sampling method. If any of the patients had the following situations, he/she would have been excluded from the research: occurrence of orthopedic lesions in the upper extremity; occurrence of any neurological disease; having visual, hearing, and/or cognitive impairments; inability to sit at least 1 hour independently on a chair; and absence in posttest evaluation.

### 2.2. Experimental Design

**Figure 1:**
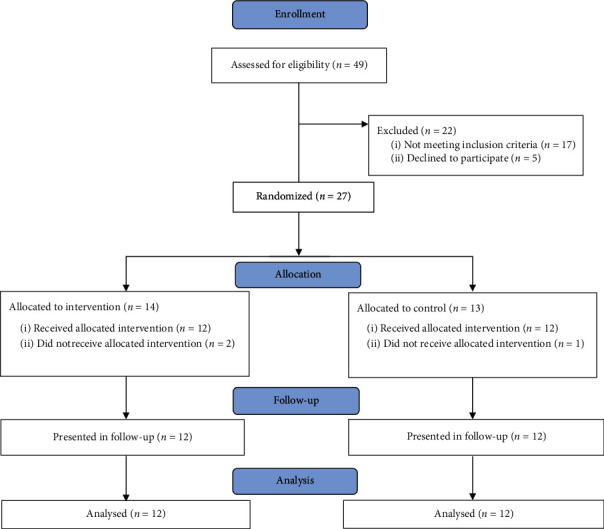
Flowchart of the participants included in the study.

This study was a double-blind randomized clinical trial with two arms. The research was done at rehabilitation clinics in Tehran. There were 49 patients with chronic stroke screened and interviewed from October 2019 to December 2020; 17 of them were excluded because of not meeting the inclusion criteria, and 5 patients declined to participate (Figure 1). The eligible participants were allocated into two groups: AOT (*n* = 13) and control group (*n* = 14) by stratified block randomization regarding positive/negative motor evoked potential (MEP) in abductor policis brevis (APB) and also the side of stroke. The randomization was administered by an epidemiologist unaware of the study with the use of Excel software. It has to be mentioned that in this study, patients and the assessors were blind to the group allocations.

### 2.3. Outcome Measures

In this study, the COPM was considered as the primary outcome measure and ARAT, BBT, MEP, and Actual Task Performance Assessment were selected as secondary outcome measures. All clinical assessments were administered in a constant order by a trained occupational therapist with a 10-year experience. An expert in physical medicine and rehabilitation specialized in the use of transcranial magnetic stimulation (TMS) assessed the MEP in a separate session from other evaluations.

#### 2.3.1. Canadian Occupational Performance Measure (COPM)

This measurement has been used in a semi-structured interview to identify patients' main concerns in the occupational areas including self-care, productivity, and leisure/play on a 0–10-point self-rating scale. The COPM enabled team research to identify occupational problems and measure patients' perception of their performance and satisfaction with the selected tasks before and after each intervention. The validity, reliability, and responsiveness of the COPM are reported as acceptable in many diseases such as stroke [34]. A change of two points or more on the COPM is considered clinically significant [35].

#### 2.3.2. Fugl-Meyer Assessment of Upper Extremity (FMA-UE)

FMA-UE is a stroke-specific measure of sensorimotor impairment and includes 33 items on a 3-point ordinal scale (0 = cannot perform, 1 = can partially perform, 2 = can perform fully). The summation of scores will be a maximum of 66 [36, 37]. The construct validity, inter-rater reliability, and intra-rater reliability of this scale have been reported as very good [37].

#### 2.3.3. Action Research Arm Test (ARAT)

ARAT is a 19-item scale divided into four basic movements [38] of grasp, grip, pinch, and gross movements that measures UE (arm and hand) function. It is scored with 0, 1, 2, or 3, with a total summation of 57 in which higher scores indicating better arm motor performance. The test has been reported as valid [38, 39] and sensitive to therapy-related [40, 41] and spontaneous [38, 39, 42] changes post stroke. It is a reliable and valid measure to assess upper limb functions in stroke subjects [39].

#### 2.3.4. Box and Block Test (BBT)

The BBT is frequently used as a measure of dexterity. The BBT apparatus consists of a box of specified dimensions divided into two sections. The test contains picking up a block out of a box and transferring it over a wall into the other side of the box. The total scoring is by counting the number of blocks carried over the partition from one side to the other during 60 seconds [37]. The test has been shown to be valid and reliable [43].

#### 2.3.5. Motor Evoked Potential (MEP)

One of the variables related to brain physiology and motor pathways that can be recorded by the TMS device is MEP [44, 45]. By sending pulses and currents through a magnetic coil, local neurons and consequently pyramidal cells, spinal cells, or the corticospinal tract can be stimulated. Depending on the brain stimulation area, there would be a recordable MEP at the end of the path, where the target muscles contract. The tendon muscles or finger extensors are usually used for this recording [46]. In this study, central motor conduction time (CMCT) was the analyzed finding related to the MEP.

To record the MEP using a Magstim 200 stimulator (Magstim Co. Ltd., Whiteland, Dyfed, UK), the patient had to sit in a quiet room in a special chair [45]. Cerebral cortex area M1 and appendix of the seventh cervical vertebra (C7) were selected as stimulation points for extensor indicis (EI) and abductor policis brevis (APB) as target muscles. The thumbs and index fingers play an important role in many daily activities, such as the eating tasks [47, 48].

In the case of the thumb, the abducted positions contribute to about two-thirds of the grips, and for this reason, we recorded the APB muscle, which is the main active muscle of the thumb during these positions. Also, to record MEP of the index finger, EI was selected due to its independence from other finger extensors during the excitation and palpation. There are other studies that have considered APB [49, 50] and EI [51, 52] for their MEP recording as well.

To record the MEP, about 3 to 5 waves with good reproducibility and high intensity were selected, and then, by subtracting cervical latency from the M1 latency, the CMCT was calculated [49].

#### 2.3.6. Actual Task Performance Assessment

To improve the validity of the data and the results of the interventions, a scale derived from the Chedoke Arm and Hand Activity Inventory was used as an objective assessment [32]. Scoring of the eating subtasks, which were used as training components in the study, was according to the assessor's opinion.

### 2.4. Intervention Protocol

Because the occupations and tasks for the intervention were related to eating, thereby some basic eating-related tasks were selected such as using fork, pouring water from bottle to glass, and drinking from a hard glass with the affected limb. In an expert panel consisting of one neuroscientist and four proficient occupational therapists working in neurologic rehabilitation settings, based on the evidence and expert opinions, the selected tasks were analyzed and divided to short part sequences of the whole task execution (Table 1).

It has to be mentioned that to consider the upper limb ability and progress of the participants during the sessions, some meaningful tasks with more difficulties such as pouring water from pitcher to glass, eating soup with spoon from bowl, and drinking from a soft glass with the affected limb were also provided. If a patient had adequate performance and satisfaction in performing any of the activities (I–IV) earlier than the time expected, he/she could perform the more difficult activities in the next sessions.

**Figure 2:**
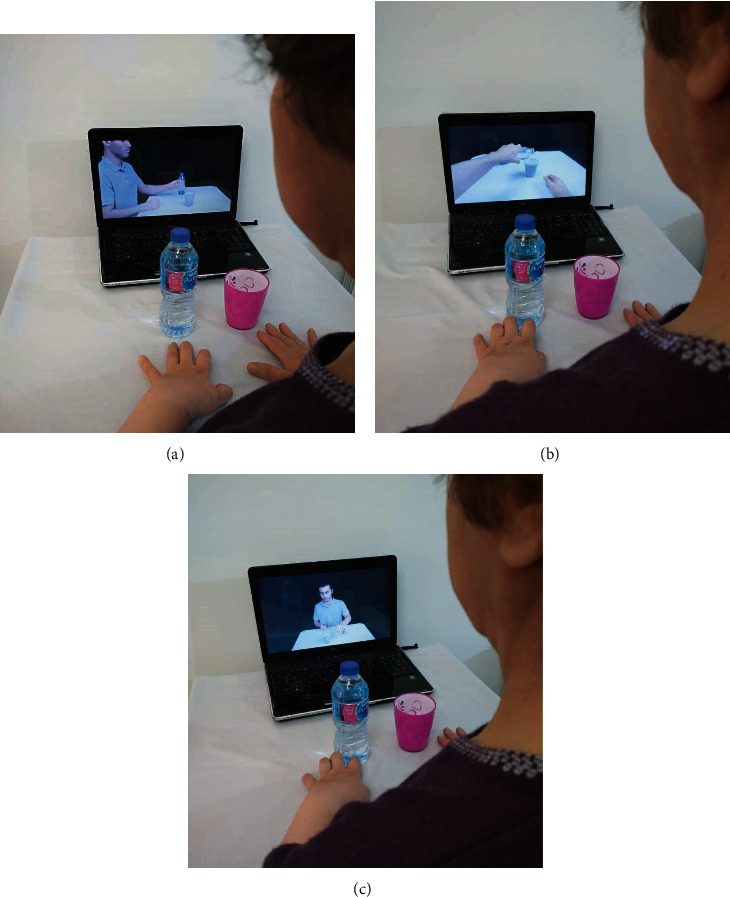
A left hemiplegic patient in AOT group watching AOT videos of (a) lateral view, (b) point of view, and (c) front view.

**Figure 3:**
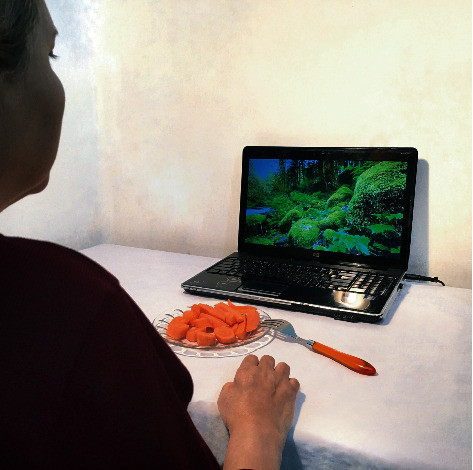
A right hemiplegic patient in control group watching landscape (sham) videos.

Afterwards, to prepare videos for AOT intervention, a Fujifilm X-H1 camera filmed those actions and tasks while acted by a young healthy model. The performance of subtasks A–H by the model was recorded from 3 angles: lateral view, point of view, and front view (Figures 2(a)–2(c)). Also, for the control group, nature and landscape videos were provided as sham observation videos (Figure 3). The quality for all videos was chosen with a 1080p resolution. After providing a final version of the edited video footages, to identify the time required for assessments and to ensure patient safety and technical considerations of the interventions mainly the AOT, a 4-week pilot study was conducted with three stroke patients.

#### 2.4.1. AOT Group

Due to motor learning theories and recent approaches [53, 54], we dedicated 3 sessions to observe and practice each task, so a total of 12 sessions were considered for the 4 tasks (3 times a week). To maintain the effects of the previous practiced task, at the end of each task practice period (after 3 sessions), at the beginning of the next sessions, the previous tasks had been viewed as a complete task for 6 minutes and then performed as a whole. For example, activity I was selected for the first 3 sessions and activity II for the next 3 sessions. Therefore, in the fourth session of the study, before observing and performing the components of activity II, the whole task observation/execution of the activity I should have been performed for 6 minutes (3 minutes observation + 3 minutes execution).

Each of the I–IV activities included functional components that were briefly explained to the patients at the beginning of each of the three intervention sessions. Each session lasted 45–60 minutes, and the steps followed in each session were as described below:

1. The video of how to perform each component (part-task) was played from 3 angles for a total of about 2 minutes; each angle was being shown approximately 3 times
2. After watching the video (action observation) of each activity component, the participant should have performed the same movements and tasks for 3 minutes. If necessary, in addition to monitoring the intervention session, the therapist provided appropriate physical assistance for the patient to complete the activity
3. Before the end of each session and after observing and performing all the components, the whole task was shown for about 3 minutes in 3 angles (i.e., each angle for 1 minute)
4. After watching the whole task video, the participants should have practiced the same movements and tasks for 3 minutes

#### 2.4.2. Control Group

Similarly, in the control group, the protocol was designed with 12 sessions, 3 days a week, and in each session about 45–60 minutes. In contrast to the AOT group, before performing eating-related tasks, the patients in this group should have observed landscape and nature videos (sham) despite the observation of the eating-related videos. All other items such as the eating-related tasks, sequences, time, and order of subtask executions in the control group were the same as the AOT group (Table 1). Activities I–IV including functional components were briefly explained to the patients at the beginning of each of the three intervention sessions. Furthermore, like the AOT group, if practicing these tasks were too easy for any patient, the more difficult tasks mentioned earlier were possible to be practiced in the following sessions.

### 2.5. Sample Size

The sample size was calculated regarding the occupational performance (COPM) as the primary outcome measure of this study. A moderate effect size was considered (*f* = 0.25 or eta − squared = 0.06) [55] due to the reported effectiveness of AOT on occupational performance [56].

With regard to the study design, using the G∗Power 3.1 statistic software, the following values were applied: *α* = 0.05, power = 80%, number of groups = 2, number of measurements = 3, correlation among repeated measures = 0.5, and nonsphericity correction = 1. Although a total sample size of 27 patients was estimated, because of many limitations during the COVID-19 pandemic and 3 dropouts, an interim analysis was done. The results showed a significant difference between two study groups for the primary outcome (COPM), and the main hypothesis was confirmed. Thereby, the patient recruitment was stopped with 24 participants who completed the follow-up (Figure 1).

### 2.6. Statistical Analysis

We calculated the descriptive and analytic statistics using the software SPSS, version 16 (SPSS Inc., Chicago, IL, USA). To compare the effects of two interventions on most outcomes during the study, analyses of variance (ANOVAs) with repeated measures with a between-subject factor at 2 levels (2 groups) and a within-subject factor at 3 levels (time: before, after, and follow-up) were conducted. Also, the time × group interaction effect was analyzed. Analysis of post hoc with a Bonferroni correction was used when a significant interaction effect was detected. To investigate the repeated measure effect size, values based on partial eta squared (*η*^2^) were considered as small (=0.01), medium (=0.06), and large (=0.14) [55]. In the present study, MEP was just recorded at baseline and posttest; therefore, to analyze CMCT data before and after the interventions, we calculated change scores for each group and compared them by using the Mann-Whitney *U* test. Significance was set at 0.05.

## 3. Results

Of 27 participants who enrolled in the study, 12 subjects in the control (9 males, 3 females; mean ± SD age, 56.58 ± 11.21) and 12 subjects in the AOT (7 males, 5 females; mean ± SD age, 53.50 ± 10.55) groups completed the study and were finally considered for statistical analyses. In the intervention group, 1 participant was excluded from the study because of an arm fracture incidence. In the control group, 2 participants were excluded; the first one was because of traveling and the second one was because of not willing to go outdoor during the COVID-19 pandemic. All characteristics of the analyzed participants and the study flowchart are presented in Table 2 and Figure 1, respectively.

Both groups showed improvements in the mean score of the primary outcome measure (COPM) and most of the secondary outcome measures (FMA, ARAT, BBT, CMCT, and the actual task performance test) during the study. After 1 month of study, the changes in the mean scores were maintained and did not decrease until follow-up assessments.

The time × group effect was significant for COPM performance (*F*(1.15, 25.40) = 7.2, *p* = 0.03) and satisfaction (*F*(1.16, 25.67) = 19.3, *p* = 0.001) with large effect sizes (performance *η*^2^ = 0.26, satisfaction *η*^2^ = 0.46) (Table 3). Similarly, the actual task performance of eating significantly changed (*F*(1.25, 27.66) = 6.43, *p* = 0.04) with a large effect size (*η*^2^ = 0.26) (Table 3). In contrast, other clinical assessments such as FMA, ARAT, and BBT did not show significant differences among two treatments during the study (*p* ≥ 0.05). In contrast, other clinical assessments such as FMA, ARAT, and BBT did not show significant differences among two treatments during the study (*p* ≥ 0.05).

Comparisons of all pairs were analyzed using the Bonferroni correction test, and a significant difference between premeans and postmeans was observed (*p* < 0.05) for all the outcomes, while there was not a significant difference between post and follow-up results (*p* ≥ 0.05).

It has to be mentioned that because MEP could not be detected in some patients, the obtained CMCT was analyzed with 13 and 17 samples for APB and EI, respectively. The results of CMCT were different in APB and EI muscles (Table 4). While there was not a significant change in the CMCT of APB in the control group, this outcome measure in the AOT group showed significant improvement. In the EI muscles, CMCT has been changed in both groups, but there was not a significant difference between treatments (Table 4).

## 4. Discussion

This novel study focuses on employing the important and meaningful occupations of the clients into the AOT process, while heretofore, this method was being practiced with simple movements and activities that were not asked if they were meaningful or meaningless to the participants. As mentioned earlier, the meaningfulness can become a source of motivation and volition during the treatment process and could lead to more therapy engagement. Thereby, watching and imitating the videos of performing a contextual, individualized and real occupation could help the patients feel the sense of accomplishment and mastery over that occupation/task. Other new information that may be extracted from this research is the impact of meaningful AOT as a top-down intervention on occupational, functional, and neurophysiological outcomes. This randomized clinical trial indicated that either with or without watching meaningful and self-selected activities, practicing these activities would help chronic stroke patients to make improvements in their desired occupational performance/satisfaction and also in motor recovery and function of the affected upper limb. Although determining the superiority between meaningful and less meaningful AOT remains questionable, at first and in this stage, it was necessary to confirm the novelty and effectiveness of the meaningful AOT protocol on clinical and neurophysiological changes in the brain.

As a result of this study, both experimental and control groups showed changes in COPM, FMA, ARAT, and BBT significantly. Although CMCT of the EI was improved in both groups, the APB just showed significant changes in the AOT group. The main findings of this research were the significant differences between groups which were only seen in COPM, actual task performance assessment, and CMCT of the APB.

In this study, COPM besides the actual performance assessment was used to evaluate occupational participation and also to set the treatment protocol. Since this study is the first AOT trial using these assessments, the comparison with other AOT studies would be just feasible regarding other participation outcomes. Similar studies have reported that improvement in Stroke Impact Scale (SIS), Functional Importance Measure (FIM), and Barthel Index (BI) was more significant through watching a model's action execution during AOT in comparison with watching landscape or sham videos as control treatment [11, 50, 57, 58]. In another study, this advantage of AOT was also seen over the mirror therapy method [59]. In contrast to the above results, a study claimed that there was no significant difference between the changes in FIM scores due to AOT and control treatment [20]. The possible reason for this might be the short period of each AOT session. The time for watching videos in that study was reported to be about 15 minutes each session, in contrast to 45–60 minutes devoted to AOT sessions in the present study.

Regarding CMCT, related changes in APB as a result of AOT showed significant advantages over the control group. This probably means that watching a video of meaningful activities can increase the effect of execution of those activities on the excitability of the cortical-spinal pathway and decrease the time of transmitting motor commands from M1 to the tendon muscle (APB). Only two similar studies have investigated the changes in MEP due to AOT poststroke [50, 60]. In a randomized control trial, results of CMCT showed a significant decrease for APB within and between groups [50]. Although it studied acute patients and lasted for 8 weeks, the results were in agreement with the present study. In another study, the results could not show a significant change in CMCT of APB as after AOT [60]. Many of neurophysiological research use APB to record MEP [50, 60], but there are scarce studies that investigated the EI [52]. Because the extensor muscles play an important role in many activities, in this study, it was decided to examine the EI beside the APB to observe the impact of AOT on CMCT. Although both groups had significant improvements, between-group changes were not considerable. This result could be a subject of future research.

In the present study, although motor recovery of upper limb (reflected by FMA) after AOT was more than after control treatment, the difference did not reach a significant statistical level between groups. The results might be more significant if a larger sample was recruited or the therapy sessions would be continued. Some studies have reported that the motor recovery of impaired upper limb was significantly higher due to AOT compared to control [11, 50, 57, 61]. It has to be mentioned that in the present study, the focus of AOT in chronic patients was on meaningful and complex activities rather than basic movements. In contrast, all of the mentioned studies (1) were investigated in acute stroke patients (<6 months poststroke) and (2) used various tasks from simple to complex for the observation and execution process. Therefore, it might be argued that the earlier the treatment and the more basic the movements, the better the motor recovery.

The results of the upper extremity function in this study (reflected by ARAT and BBT) did not show statistically meaningful changes between groups. In contrast to the present study considering ARAT, in an 8-week AOT study, advantage over control treatment was reported [50]. There are also controversial results related to BBT after AOT [20, 59, 61].

Overall, this study indicates that action observation and execution of meaningful activities as a MNS-based technique can enhance the performance/satisfaction of the selected occupation in chronic stroke patients more than just executing those activities. Meaningful AOT can also improve cortical excitation; therefore, it probably provides the brain neural networks for a higher chance of persistent plasticity.

## 5. Limitations

In this study, to make the situation more meaningful and realistic for the participants, some activities such as eating a daily meal (rice or soup) were tried to be practiced in a lunchtime session. Therefore, fixing the session times and avoiding overlaps between lunchtime sessions was a challenge during the study. However, this problem was also solved by the cooperation of subjects and setting a dynamic weekly time schedule for the sessions.

The COVID-19 pandemic slowed the process of patient recruitment for the study. Disease recurrent waves, in addition to national lockdowns, made the brain-mapping laboratory located in the hospital isolated and semi-closed. However, these restrictions were managed by planning the time for patients to enter the study in nonpeak periods of the pandemic, as well as holding a few sessions at the patients' homes when it was not possible to attend the clinic, and most importantly, by observing health protocols during the sessions.

## 6. Recommendations

- Implementing a study with more subjects to provide the sample size needed for examining secondary variables of this study, such as corticospinal excitability
- Comparing the effectiveness of AOT of meaningful and selected activities with AOT of nonselected and less meaningful activities, as well as comparing these two with a combination of them
- Using patients themselves and their healthy limbs as models in producing videos of activities through graphic technologies
- Investigating the method of meaningful action observation in the form of novel technologies such as virtual reality (VR) systems that can provide patients with various environments and meaningful activities
- Studying the effects of simple observation of the activities without execution in patients with very low motor function as an adjunct therapy in the early stages of stroke

## 7. Conclusion

Meaningful action observation training could possibly enhance the effects of activity/occupation-based interventions on occupational performance and satisfaction, as well as cortical-spinal excitability. This method of AOT seemed to be innovative, client-centered, and affecting neuroplasticity. However, it might not make much difference in improving the effectiveness of activity/occupation-based interventions on upper limb motor recovery and functions of the impaired upper limb in patients with chronic stroke.

## Figures and Tables

**Table 1:** Eating-related meaningful tasks (I–IV), their subtasks, and the procedure time.

Task						
Subtask	Task I Pour water from bottle to glass	Task II Eating food bimanually	Task III Drink water from a hard glass	Task IV Eat a piece of carrot with fork	*Time for watching films (AOT* ^∗^ *or sham* ^∗∗^)	*Time for performance*
A	Reach to the bottle	Reach to the spoon and fork	Reach to the glass	Reach to the fork	2 m	3 m
B	Grasp the bottle	Grasp the spoon and fork	Grasp the glass	Grasp the fork	2 m	3 m
C	Bring the bottle near to the glass	Take some meal	Bring the glass near to the mouth	Bring the fork near to the carrot	2 m	3 m
D	Pour water into glass	Bring spoon to mouth and eat	Drink from the glass	Bring the carrot to the mouth	2 m	3 m
E	Reach out to desktop	Reach out and place spoon and fork beside the plate	Reach out to the desktop	Reach out to the dish	2 m	3 m
F	Release the bottle	Release the spoon and fork	Release the glass	Release the fork	2 m	3 m
G	Rest arm	Rest arm	Rest arm	Rest arm	2 m	3 m
H	Whole task	Whole task	Whole task	Whole task	2 m	3 m

^∗^Films in AOT includes videos of a model performing the A-H subtasks for intervention group. ∗∗ Films in sham include videos of nature and landscapes for control group. *m: minute; whole task: the combination of subtasks A–G.*

**Table 2:** Distribution of demographic variables in the intervention and control groups.

Qualitative variables	Intervention (*n* = 12)	Control (*n* = 12)
Sex	*n* (%)	*n* (%)
Female	5 (41.7%)	3 (25%)
Male	7 (58.3%)	9 (75%)
Type of stroke		
Hemorrhagic	4 (33.3%)	2 (16.7%)
Ischemic	8 (66.7%)	10 (83.3)
Affected side		
Right	5 (41.7%)	6 (50%)
Left	7 (58.3%)	6 (50%)
Handedness		
Right	11 (91.7%)	10 (83.3%)
Left	1 (8.3%)	2 (16.7%)
Upper arm Brunnstrom stage		
III	3 (25%)	4 (33.3%)
IV	6 (50%)	2(16.7%)
V	3 (25%)	6 (50%)
Hand Brunnstrom stage		
III	4 (33.3%)	4 (33.3%)
IV	6 (50%)	3 (25%)
V	2 (16.7%)	5 (41.7%)
Motor evoked potential (APB)		
+	6 (50%)	7 (58.3%)
−	6 (50%)	5 (41.7%)
Quantitative variables	Intervention (*n* = 12)	Control (*n* = 12)
	Mean ± SD	Mean ± SD
Age (year)	53.5 ± 10.55	56.58 ± 11.21
Time since stroke (month)	39.75 ± 28.35	42.58 ± 29.25

**Table 3:** Pre, post, and follow-up outcome measures of the intervention and control groups (time × group interaction).

	Baseline		Post (at 4 weeks)		Follow-up (at 8 weeks)					
	Mean ± SD		Mean ± SD		Mean ± SD					
	Intervention (*n* = 12)	Control (*n* = 12)	Intervention (*n* = 12)	Control (*n* = 12)	Intervention (*n* = 12)	Control (*n* = 12)	Wilks lambda	*F*	*P* − *v*	Effect size (Eta^2^)
COPM (performance)	3.00 ± 1.34	3.41 ± 2.35	6.58 ± 2.02	5.29 ± 2.43	6.50 ± 1.97	5.37 ± 2.44	0.73	3.83	0.03^∗^	0.26
COPM (satisfaction)	2.75 ± 1.76	3.66 ± 2.60	6.58 ± 2.46	4.83 ± 2.48	6.66 ± 2.38	4.91 ± 2.39	0.50	10.36	0.001^∗^	0.49
Actual task performance	19.41 ± 13.96	23.08 ± 11.07	29.00 ± 15.67	28.75 ± 11.81	29.00 ± 15.40	28.08 ± 11.95	0.73	3.71	0.042^∗^	0.26
FMA-UE	38.66 ± 12.57	41.33 ± 14.53	46.16 ± 7.1	46.41 ± 12.0	46.33 ± 14.72	46.66 ± 12.68	0.91	0.96	0.39	0.08
ARAT	23.08 ± 24.18	25.83 ± 19.78	27.00 ± 23.41	30.16 ± 20.70	26.83 ± 23.53	29.58 ± 20.76	0.96	0.41	0.66	0.03
BBT	9.82 ± 12.22	12.38 ± 10.45	13.16 ± 15.48	13.46 ± 10.95	13.08 ± 15.52	13.25 ± 11.19	0.88	1.37	0.275	0.11

*Note:* ^∗^ = statistically significant; COPM: Canadian Occupational Performance Measure; *F*: test for repeated measures 2-way ANOVA; FMA-UE: Fugl-Meyer Assessment (upper extremity); ARAT: Action Research Arm Test; BBT: Box and Block Test.

**Table 4:** MEP comparison between the intervention and control groups, before and after the therapy.

	Baseline Mean ± SD		Post (at 4 weeks) Mean ± SD		Mean change ± SD		Mann-Whitney test	*P*-value
CMCT (APB)	Intervention (*n* = 6)	Control (*n* = 7)	Intervention (*n* = 6)	Control (*n* = 7)	Intervention (*n* = 6)	Control (*n* = 7)		
	12.41 ± 3.15	13.24 ± 2.05	10.20 ± 2.20	13.24 ± 1.68	−2.21 ± 2.32	0.00 ± 1.01	−2.29	0.022^∗^

CMCT (EI)	Intervention (*n* = 7)	Control (*n* = 10)	Intervention (*n* = 7)	Control (*n* = 10)	Intervention (*n* = 7)	Control (*n* = 10)	−0.48	0.625
	12.38 ± 4.10	14.60 ± 4.59	11.42 ± 2.00	13.75 ± 3.40	−0.96 ± 2.04	−0.84 ± 2.88		

^∗^ = statistically significant; MEP: motor evoked potential; CMCT: central motor conduction time; APB: abductor policis brevis; EI: extensor indicis.

## Data Availability

The data gathered in this research could be reached on request from the corresponding author.
